# Quality Indicators for Avalanche Victim Management and Rescue

**DOI:** 10.3390/ijerph18189570

**Published:** 2021-09-11

**Authors:** Alexandre Kottmann, Mathieu Pasquier, Giacomo Strapazzon, Ken Zafren, John Ellerton, Peter Paal

**Affiliations:** 1 Rega—Swiss Air Ambulance, Zürich Airport, 8058 Zürich, Switzerland; 2 Emergency Department, Lausanne University Hospital, 1011 Lausanne, Switzerland; Mathieu.Pasquier@chuv.ch; 3 International Commission for Mountain Emergency Medicine (ICAR MedCom), 8058 Zürich, Switzerland; Giacomo.strapazzon@eurac.edu (G.S.); kenzafren@gmail.com (K.Z.); johnellerton01@btinternet.com (J.E.); peter.paal@bbsalz.at (P.P.); 4 Institute of Mountain Emergency Medicine, Eurac Research, 39100 Bolzano, Italy; 5 CNSAS—Corpo Nazionale Soccorso Alpino e Speleologico, National Medical School, 20124 Milano, Italy; 6 Alaska Native Medical Center, Department of Emergency Medicine, 4300 Diplomacy Drive, Anchorage, AK 99508, USA; 7 Stanford University Medical Center, Department of Emergency Medicine, 900 Welch Road, Palo Alto, CA 94304, USA; 8 Department of Anaesthesiology and Intensive Care Medicine, St. John of God Hospital, Paracelsus Medical University, Kajetanerplatz 1, 5020 Salzburg, Austria

**Keywords:** avalanche, quality indicator, quality improvement, resuscitation, emergency medicine, emergency medical services, rescue, consensus process

## Abstract

Decisions in the management and rescue of avalanche victims are complex and must be made in difficult, sometimes dangerous, environments. Our goal was to identify indicators for quality measurement in the management and rescue of avalanche victims. The International Commission for Mountain Emergency Medicine (ICAR MedCom) convened a group of internal and external experts. We used brainstorming and a five-round modified nominal group technique to identify the most relevant quality indicators (QIs) according to the National Quality Forum Measure Evaluation Criteria. Using a consensus process, we identified a set of 23 QIs to measure the quality of the management and rescue of avalanche victims. These QIs may be a valuable tool for continuous quality improvement. They allow objective feedback to rescuers regarding clinical performance and identify areas that should be the foci of further quality improvement efforts in avalanche rescue.

## 1. Introduction

Decisions in the medical management and rescue of avalanche victims are complex. They must be taken in pre-hospital environments with objective dangers, where risk assessment is necessary, usually with time constraints. Guidelines have been developed for the resuscitation and on-site triage of avalanche victims, whether completely buried with the head below snow and at risk of asphyxia or partially buried, with the head out of the snow and not at risk of asphyxia [1,2]. Adherence to these guidelines is variable and may depend on the case load of individual emergency medical service agencies (EMS) [3,4,5,6]. The collection of specific avalanche-related information is necessary to guide the prehospital medical management. However, their documentation might be challenging due to the inherent challenges of an avalanche scene. Incomplete medical records, missing specific avalanche-related information, are a recurrent limitation that may impair the transfer of care at the hospital. This could negatively impact outcomes as well as limit the retrospective evaluation of on-site management [3,5]. There is a need to improve the quality of the management and rescue of avalanche victims.

The first step in quality improvement is to identify quality indicators (QIs). This allows for the measurement and monitoring of quality by a health care system. QIs also enable comparisons amongst health care systems. In this study, our aim was to identify QIs for the management and rescue of avalanche victims, using a consensus process method.

## 2. Materials and Methods

We conducted the study between October 2017 and October 2018. We used a five-round modified nominal group consensus process to identify the most relevant QIs [7,8]. The selection of potential QIs started with brainstorming. We invited all members of the International Commission for Mountain Emergency Medicine (ICAR MedCom) to propose potential QIs for the management of avalanche victims. We considered QIs to be data points that could help measure the quality of avalanche victim management and rescue by a helicopter emergency medical service (HEMS) or a terrestrial rescue team staffed with advanced health care providers that could have an impact on outcomes. The project group assessed these QIs for usability according to the National Quality Forum Measure Evaluation Criteria and adopted the necessary revisions [9]. The project group identified additional potential QIs by analyzing existing guidelines [1,10,11]. This process resulted in a list of QIs, which we used for the consensus process.

We invited all members of the ICAR MedCom with clinical or scientific expertise in the management and rescue of avalanche victims to participate in the expert panel. Additionally, all members of the ICAR MedCom were asked to propose potential additional experts outside of the ICAR MedCom. The modified nominal group technique consisted of four email rounds followed by a consensus meeting. To reduce potential bias, the experts were blinded to each other’s identities during the email rounds. In the first round, experts were asked to suggest modifications of the proposed QIs as well as to propose new QIs. In the second round, experts were provided with the original, the new, and the modified QIs from the first round. They were asked to rate the importance of each proposed QI for measuring the quality of avalanche victim management and rescue using a 5-point Likert rating scale (ranging from 1 = not at all important to 5 = very important). QIs rated ≥4 by >75% of the experts were included in the third round, in which the experts had to choose the 25 most important QIs [12]. In the fourth round, the experts had to rank the top 25 QIs in order of importance. The fifth round consisted of a consensus in-person meeting, in which the final set of Qis were chosen. 

Calculations were performed with Excel (Microsoft Corporation, Redmond, WA, USA). No medical records were required for this project. The Vaud, Switzerland cantonal ethical commission for research exempted the study from the formal approval process (CER-VD, Req-2018-00489).

## 3. Results

**Figure 1:**
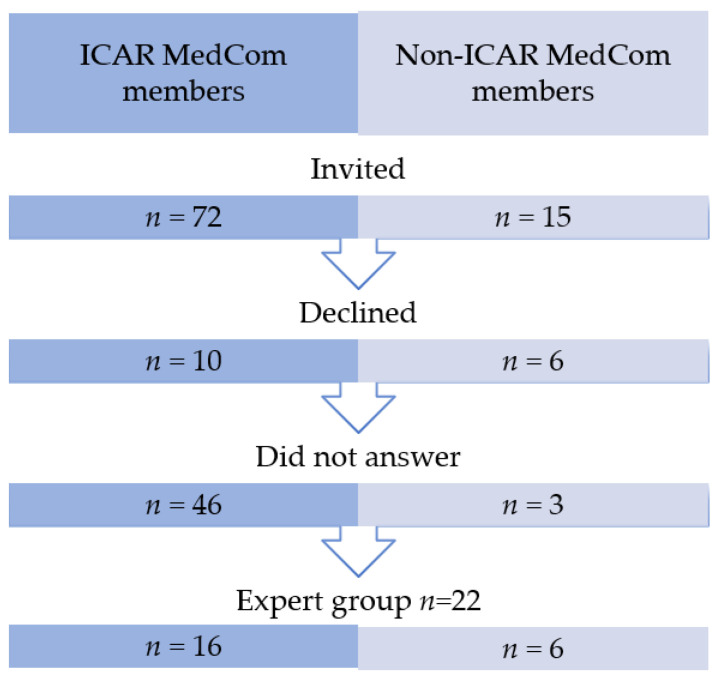
Selection of the expert group.

**Figure 2:**
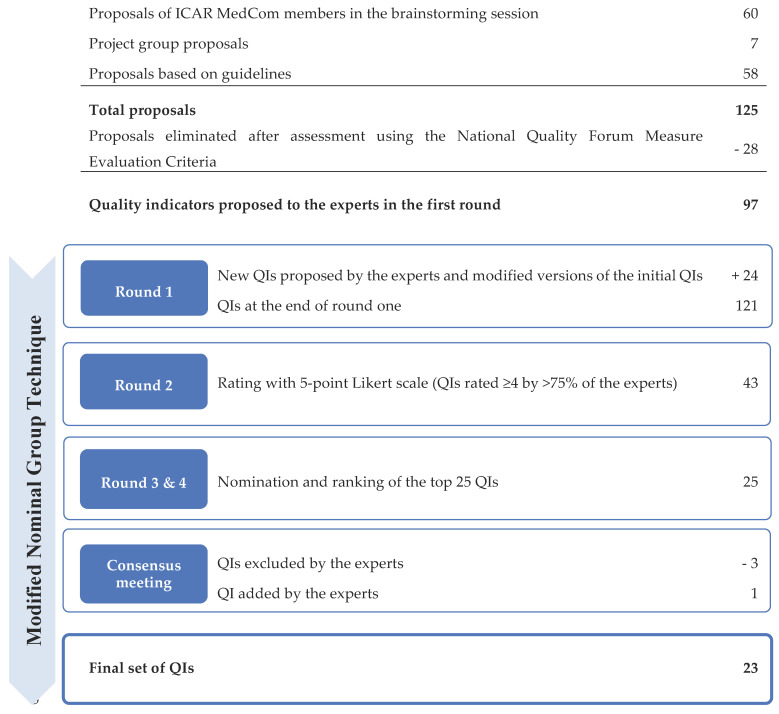
Identification and selection process for quality indicators (QIs).

We invited 72 members of ICAR MedCom and 15 non-members to participate in the study. Twenty-two experts agreed to join the expert group (Figure 1 and Supplementary File S1). Brainstorming by the ICAR MedCom members and analysis of the existing recommendations by the project group yielded 125 proposed QIs. After assessment of the QIs using the National Quality Forum Measure Evaluation Criteria and elimination of duplicate proposals, 97 proposed QIs were included in the consensus process and proposed to the expert group at the first round. The number of QIs at each step is shown in Figure 2. A list of the 121 QIs at the end of the first round is shown in Supplementary File S2. 

During the consensus meeting, the experts made minor modifications to the definitions and wordings of 15 QIs, excluded 3 proposed QIs, and added 1 QI (Supplementary File S3). The final set was composed of 23 QIs (Table 1). The QIs were divided into five categories: prior to avalanche rescue mission (*n* = 4), patient assessment (*n* = 5), patient management (*n* = 9), transport (*n* = 1) and in-hospital management (*n* = 4). The minimum data required to measure the 23 QIs are shown in Supplementary File S4.

## 4. Discussion

Defining and measuring QIs for the management and rescue of avalanche victims are important steps towards the uniform collection of key data on avalanche rescue. This should allow for the better comparison of studies from a variety of EMS agencies. Research in the management and rescue of avalanche victims is limited by low incidence, and by the heterogeneity, quality, and completeness of data from various rescue services. A comprehensive template for uniform data collection and the reporting of avalanche rescue missions should be developed in the future [11,13,14]. 

By combining brainstorming amongst key stakeholders with the analysis of recent recommendations and guidelines, and a consensus process amongst an international group of experts, we attempted to combine the advantages of multiple methods for the development of QIs, including email rounds using the Delphi technique, structured rating and prioritisation using the nominal group technique, and finally, a consensus conference to discuss and finalise the QIs [8]. 

### 4.1. Prior to Rescue

Time intervals, such as the time between the alarm at the dispatch centre and the arrival of the first rescue team on scene, vary widely amongst EMS agencies (QIs 1 and 2) [15]. A reduction in these time intervals is critical to reducing the burial duration of completely buried avalanche victims (QI 3).

In an avalanche accident, most completely buried victims die from asphyxia. The duration of burial determines the probability of survival, which drops dramatically from 90% after 15 min to about 34% after 35 min [16,17,18]. The probability of survival can be increased dramatically by reducing the time between avalanche burial and the exposure of the face (burial time), allowing rescuers to open the airway by removing avalanche debris [19,20].

Burial time (QI 3) depends on the speed of the combination of companion and professional rescue. Although early dispatch of HEMS directly to the accident site is important [21,22], rapid companion rescue with the immediate location and extrication of a completely buried victim followed by immediate basic life support if the victim is in cardiac arrest is crucial [5]. Victims in cardiac arrest, extricated and resuscitated by companions (QI 4) have a greater probability of survival compared to those extricated and resuscitated by professional rescuers who arrive later [5]. Victims extricated later are more likely to sustain severe irreversible hypoxic damage from asphyxiation [3].

### 4.2. Patient Assessment 

In avalanche rescue, specific information, such as airway patency (QI 5), the presence of an air pocket (QI 6), ECG monitoring (QI 7), and core temperature, measured properly on site (QI 8 and 9), are crucial for decision making and treatment. This information is required at the scene and again at the admitting hospital. The acquisition and documentation of these data are included in the final set of QIs (Table 1). Core temperature tends to have greater prognostic accuracy than burial time. The Hypothermia Outcome Prediction after Extracorporeal life support (HOPE) score for hypothermic patients in cardiac arrest may be the current decision tool of choice for patients in hypothermic cardiac arrest, including avalanche victims, in hospital [23,24]. However, the HOPE score has not yet been applied specifically to avalanche victims and does not include the duration of burial or the presence or absence of an air pocket.

### 4.3. Patient Management

Avalanche victims are often in critical condition. About 30% of avalanche victims are in cardiac arrest by the time an EMS arrives [21]. Although advanced airway management is not mandatory during on-site resuscitation, there are advantages to having a secure airway during transport with ongoing advanced life support or after ROSC. Securing the airway allows better oxygenation and ventilation as well as better airway protection [25]. Advanced airway management is a complex technical skill that can be seen as a proxy for general quality of care. Safe and effective advanced airway management requires a high level of expertise and a strong quality assurance system. Successful advanced airway management in avalanche victims transported to hospital (QI 10) is closely correlated with successful intubation, a common QI measure [26].

The high quality of care for avalanche victims includes whole-body insulation (QI 16). Whole-body insulation is critical to prevent further cooling. The risk of cardiac arrest in hypothermic patients increases as core temperature decreases [27]. The immediate recognition of rescue collapse (sudden cardiac arrest) is crucial to limit the length of time without effective circulation (no flow time). Measures to prevent hypothermic cardiac arrest include whole-body insulation and gentle handling during extrication, treatment, and transport. A low rate of rescue collapse likely reflects a high quality of care (QI 11).

Both technical skills and the medical management of avalanche victims require decision making with time constraints, based on specific knowledge and information. Four of the QIs are based on the European Resuscitation Council algorithm for the medical management of avalanche victims [10]. This evidence-based algorithm guides attempted resuscitation of victims with a chance of survival and avoids futile resuscitation efforts. Unless a victim has obvious signs of death, resuscitation should be started. This allows rescuers time to gather the necessary information to decide whether to continue resuscitation efforts (QI 13). Ventilation during CPR should be emphasised, because avalanche victims suffer from asphyxia [2,19,28].

The resuscitation of a victim with a patent or unknown airway, with confirmed core temperature <30 °C or the possibility of a long burial time (>60 min) when the core temperature is unknown, is an individual QI (QI 12). Victims meeting these criteria have survived with good neurological outcomes [29]. Avalanche victims in cardiac arrest with a short burial duration are unlikely to be hypothermic. They usually have poor outcomes. The widespread belief that victims with long burial times have less chance of survival is incorrect. Victims with long burials who are not asphyxiated may be protected from brain injury by hypothermia and can have good neurological outcomes [6,30]. Resuscitation efforts should be withheld, according to BLS guidelines, if the victim is not hypothermic (QI 14) or if the victim has had a long burial with obstructed airway and asystole (QI 15). 

Despite the availability of specific algorithms, a surprisingly low adherence to guidelines is common [3,6]. Incomplete prehospital documentation may be a contributing factor to poor adherence to guidelines in the field and in the hospital [3,6]. Careful documentation is essential for decision making following dedicated guidelines (QI 18). The Avalanche Victim Resuscitation Checklist (AVRC) was developed to increase the completeness of documentation and adherence to guidelines [31,32]. The expert panel identified the use of the AVRC as a support tool for documentation and decision making (QI 17). 

### 4.4. Transport and In-Hospital Management

Patients in, or at risk of, hypothermia-induced cardiac arrest should be transported directly to an ECLS centre (QI 19) [1,10,11]. In the hospital, appropriate rewarming should be initiated and continued (QI 20 and 22) [2,10,11]. Avalanche victims in cardiac arrest should be rewarmed to a core temperature of 32 °C without the return of spontaneous circulation before being declared dead. Serum potassium should be measured (QI 21) to provide guidance according to a validated outcome score, such as the HOPE score, for hypothermia victims in cardiac arrest rewarmed with ECLS (QI 23) [23].

### 4.5. Limitations

An inherent limitation of any consensus process is that the results may not be reproducible. The results depend on the composition of the expert group and could be different during multiple iterations, even with the same experts. The selection of the experts might also be biased. To reduce potential bias, we enlarged the expert panel beyond ICAR MedCom members by inviting experts from the professional networks of the ICAR MedCom members. Because our focus was on the medical aspects of avalanche rescue, we did not include technical mountain rescuers on the expert panel. This might have added other enriching points of view. The QIs we identified mainly concerned the process and outcome categories [33]. However, it is possible that other types of QIs, for example, QIs concerning training and the maintenance of skills in the structure category, might also be helpful. Because evidence and guidelines are rapidly evolving, the QIs may require modification in the future.

## 5. Conclusions

Using a consensus process, we identified a set of 23 QIs to measure the quality of management and rescue of avalanche victims. These QIs may be valuable tools for continuous quality improvement. They allow objective feedback to be given to rescuers regarding clinical performance and identify areas that should be the foci of further quality improvement efforts in avalanche rescue.

## Figures and Tables

**Table 1:** Quality indicators (QIs) for avalanche victim management and rescue (*n* = 23) selected by the expert group at the end of 5 rounds.

QI Nr	Indicator	Definition
	**Prior to the Avalanche Rescue Mission**	
QI 1	Time between alarm at dispatch centre and arrival on scene (BS)	Time between emergency call at the dispatch centre and the arrival of the first organised rescue team on scene.
QI 2	Time between accident and arrival on scene (BS)	Time between the accident and the arrival of the first organised rescue team on scene.
QI 3	Burial time [10,11] (BS)	Time between the avalanche accident and the exposure of the face.
QI 4	CPR by bystanders [10]	Proportion of victims without signs of life at extrication, for whom CPR was performed by companions, bystanders or first responders (e.g., ski patrollers) just after extrication.
	**Patient Assessment**	
QI 5	Airway patency [10,11]	Proportion of long (>60 min) complete burial (USA-critical burial) victims whose airway patency was assessed at face exposure.
QI 6	Documentation of air pocket [1]	Proportion of completely buried victims for whom the existence of an air pocket was documented and reported (air pocket: airways free of snow AND any space in front of mouth and nose).
QI 7	ECG monitoring [11]	Proportion of victims without signs of life and who did not have clear signs of death *, for whom ECG monitoring was performed on site.
QI 8	Site of temperature measurement [10]	Proportion of correct site of core temperature measurement (epitympanic in non-intubated victims not in cardiac arrest; esophageal in victims in cardiac arrest and/or intubated), amongst all victims who required temperature measurement, or when temperature was measured.
QI 9	Temperature measurement on scene [10]	Proportion of victims whose core temperature was measured at the avalanche site compared to avalanche victims for whom core temperature should have been measured.
	**Patient Management**	
QI 10	Airway management [10]	Proportion of transported victims who successfully underwent advanced airway management when it was attempted.
QI 11	Occurrence of rescue collapse (EXP)	Proportion of patients who developed cardiac arrest during extrication and transport to hospital (i.e., until hospital admission).
QI 12	Long-burial CPR start [11]	Proportion of victims without signs of life and without evident signs of death * with temperature <30 °C or burial time >60 min and patent or unknown airway for whom resuscitation (CPR) was initiated.
QI 13	Chest compression and ventilation during resuscitation [11]	Proportion of avalanche victims in cardiac arrest who received CPR, including ventilation by the rescue team (except for victims with long burial AND obstructed airway).
QI 14	Short-burial termination of CPR [10]	Proportion of victims with core temperature >30 °C AND asystole AND absence of reversible causes of cardiac arrest, for whom CPR was terminated according to guidelines (only for avalanche accidents with one victim in cardiac arrest).
QI 15	Long-burial AND termination of CPR [10]	Proportion of victims with burial time >60 min AND asystole AND obstructed airway, for whom CPR was terminated or withheld.
QI 16	Insulation [10,11] (BS)	Proportion of hypothermic victims insulated with whole-body insulation.
QI 17	Use of avalanche checklist [1](RGP)	Proportion of victims without signs of life for whom an “avalanche victim resuscitation checklist” was filled out during the prehospital phase and transmitted to the hospital team at handover.
QI 18	Completeness of documentation (RGP)	Proportion of interventions where all required information was documented (i.e., burial time, vital signs, airway patency if required, air pocket if required, ECG if required, core temperature if required, and serum potassium if required).
	**Transport**	
QI 19	Adequate transport to ECLS [11]	Proportion of hypothermic patients transported to an ECLS centre according to guidelines.
	**In-hospital Management**	
QI 20	Appropriate rewarming [10,11]	Proportion of hypothermic victims who received appropriate in-hospital rewarming.
QI 21	Serum potassium [10]	Proportion of victims for whom serum potassium was measured when recommended by guidelines.
QI 22	Hospital rewarming [10,11]	Proportion of hypothermic victims in cardiac arrest with a patent or unknown airway who were rewarmed to a core temperature >32 °C before a decision about declaration of death was made.
QI 23	Adequate patients to ECMO or CPB (EXP)	Proportion of victims in CA who received ECMO or CPB (ECLS) therapy according to guidelines.

BS brainstorming; CA: cardiac arrest; CPB: cardiopulmonary bypass; CPR: cardiopulmonary resuscitation; ECG: electrocardiogram; ECLS: extracorporeal life support; ECMO: extracorporeal membrane oxygenation; RGP research group proposal; EXP: proposed by experts during the first round. * Clear signs of death include airway obstructed with packed snow AND burial time > 60 min AND asystole, decapitation, whole body frozen.

## Data Availability

Not applicable.

## Supplementary Materials

The following are available online at https://www.mdpi.com/article/10.3390/ijerph18189570/s1: Supplementary File S1: Expert panel participating in the identification of quality indicators for avalanche victim management and rescue. Supplementary File S2: Comprehensive list of the quality indicators for avalanche victim management and rescue evaluated by the expert group during the modified nominal group technique consensus process. Supplementary File S3: Quality indicators selection done by the expert group during the consensus meeting of the modified nominal group technique. Supplementary File S4: Data required to calculate the quality indicators (QIs) for avalanche victim management and rescue.
