# Supplementary material for: Quality Indicators for Avalanche Victim Management and Rescue
Source: Int J Environ Res Public Health. 2021 Sep 11;18(18):9570. doi: 10.3390/ijerph18189570 (PMC8464975; doi:10.3390/ijerph18189570)
Supplement: Supplementary file 1 [file ijerph-18-09570-s001.zip › ijerph-1329297-supplementary.pdf]

Supplementary File S1: Expert panel participating in the identification of quality indicators for avalanche victim management and rescue.

ICAR MEDCOM EXPERTS		
**Günther Sumann**	**Physician**	Austria
Hermann Brugger	Physician	Italy
Iñigo Soteras	Physician	Spain
Oliver Reisten	Physician	Switzerland
Grégoire Zenruffinen	Physician	Switzerland
Kazue Ooshiro	Physician	Japan
Dider Moens	Physician	Belgium
Julia Fieler	Physician	Norway
Marc Blancher	Physician	France
Urs Wiget	Physician	Switzerland
Noriyoshi Ohashi	Physician	Japan
Sylweriusz Kosinski	Physician	Poland
François Albasini	Physician	France
Alison Sheets	Physician	USA
Pranav Koirala	Physician	Nepal/USA
Rowena Christiansen	Physician	Australia
**INVITED EXPERTS**		
Christopher Van Tilburg	Physician	USA
Colin Grissom	Physician	USA
Will Smith	Physician	USA
Scott Mcintosh	Physician	USA
Oliver Kreuzer	Paramedic	Switzerland
Albert R. Wheeler	Physician	USA

Supplementary File S2: Comprehensive list of the quality indicators for avalanche victim management and rescue evaluated by the expert group during the modified nominal group technique consensus process.

bs: brainstorming; **rgp**: research group proposal; **exp:** proposed by experts during the first round.

ALS: advanced life support; **BLS:** basic life support; **CA**: cardiac arrest; **CPB**: cardiopulmonary bypass; **CPC:** Cerebral Performance Category **CPR**: cardiopulmonary resuscitation; **ECG:** electrocardiogram; **ECLS:** extracorporeal life support; **ECMO**: extracorporeal membrane oxygenation; **HEMS:** helicopter emergency medical service; **ICAR:** International Commission for Alpine Rescue; **ROSC:** return of spontaneous circulation

QI Nr	QI name	QI definition
	**Prior to the avalanche rescue mission**	
1	Alarm to rescue start time (bs)	Time between the alarm being raised and the beginning of the rescue (i.e. take off of the first helicopter or start of the first terrestrial rescue team).
2	Response time (bs)	Time between the alarm being raised and arrival of the first organized rescue team on scene (helicopter, terrestrial, or ski patrol).
3	Accident to on scene time (bs)	Time between the accident and the arrival of the first organized rescue team on scene (helicopter, terrestrial, or ski patrol).
4	Burial time [1] [2] (bs)	Time from the avalanche accident until exposure of the face.
5	CPR by comrades [2]	Rate of victims in cardiac arrest at extrication, for whom CPR was initiated and performed correctly by comrades or first responders (ski patrol) directly after extrication.
6	ROSC by comrades (bs)	Rate of victims with ROSC (vital signs / signs of life) after resuscitative efforts only made by comrades.
7	Rescuer care level (bs)	Rate of ALS providers on scene compared to the total number of ALS and BLS providers on site.
8	ALS on site (bs)	Rate of avalanche rescue missions with at least one ALS provider on site.
9	Medical commander on scene (bs)	Rate of avalanche rescue missions involving multiple victims, with a dedicated medical commander on site (a person on scene coordinating the medical part of the rescue: triage, treatment, and transport to hospitals).
10	Senior Backup available on scene (bs)	Rate of avalanche rescue missions with a senior physician or paramedic available on scene as backup for questions.
10A	Senior Backup available on call (bs)	Rate of avalanche rescue missions with a senior physician or paramedic available "on call" (phone or radio) as back up for questions.
11	Training frequency med (bs)	Number of training scenarios for medical on-site management of avalanche victims per provider during a given period of time (e.g. 1 year).
12	Avalanche rescue training frequency (bs)	Number of training scenarios for the location and/or extraction of a fully buried avalanche victim per provider during a given period of time (e.g. 1 year).
13	Clinical exposure to avalanche rescue missions (bs)	Number of avalanche rescue missions for a provider of the rescue service (e.g. paramedic or physician) during a given period of time (e.g. 1 year).
14	Clinical exposure to avalanche rescue missions (base) (bs)	Number of avalanche rescue missions for a HEMS base during a given period of time (average, time period 1st January to 31st December of a given year).
15	Clinical exposure to avalanche rescue missions (HEMS) (bs)	Number of avalanche rescue missions for a HEMS organisation during a given period of time (average, time period 1st January to 31st December of a given year).
16	Clinical exposure to avalanche victims (rgp)	Total number of avalanche victims treated by a given provider in the pre-hospital setting.
17	Clinical exposure to avalanche victims in cardiac arrest (rgp)	Total number of avalanche victims in cardiac arrest treated by a given provider.
106	Accident to alarm time (exp)	Time from the avalanche accident until the alarm call by a bystander; indicator for actions already taken before calling.

	**Patient assessment**	
18	Extrication after complete burial within 90 min [1]	Rate of completely buried victims extricated within 90 minutes after burial.
107	Extrication after complete burial within 30 min (exp)	Rate of completely buried victims extricated within 30 minutes after burial.
19	Extrication after short burial [2]	Rate of victims buried <60 minutes who were extricated rapidly (prioritising speed of extrication as opposed to gentle extrication).
20	Extrication by long burial [2]	Rate of victims buried >60 minutes, who were extricated gently (prioritising gentle extrication to prevent an circumrescue collapse).
21	Burial degree / grade of burial documentation [2]	Rate of victims for whom the burial degree (grade of burial) was documented (complete - i.e. head and chest below snow vs. partial - i.e. head and chest out of the snow).
108	Burial depth documentation (exp)	Rate of victims for whom the burial depth was documented.
22	Airway patency [1] [2]	Rate of victims of long (>60 min) complete burial for whom airway patency was assessed at the time the face was exposed.
23	Air pocket documentation [3]	Rate of completely buried victims for whom the existence of an air pocket was reported (air pocket: airways free of snow AND any space in front of mouth and nose).
24	Size of air pocket [3]	Rate of completely buried victims with a documented air pocket for whom the dimension of the air pocket was reported.
25	Signs of life check [1] [2]	Rate of victims of long (>60 min) complete burial without signs of life, who were checked for signs of life for at least 1 min.
26	ECG monitoring [1]	Rate of victims who did not have clear signs of death for whom ECG monitoring was performed in the prehospital setting. Clear signs of death include airway obstructed with packed snow, burial time >60min and asystole, decapitation, or whole body frozen.
27	ECG monitoring [2]	Rate of victims in cardiac arrest for whom ECG monitoring was performed to confirm cardiac arrest or cardiac rhythm.
28	Low reading core temperature thermometer used (bs)	Rate of use of a thermometer able to measure core temperature <32°C (evt <35°C).
29	Temperature measurement on site [2]	Rate of victims for whom core temperature was measured at the site of the avalanche.
30	Temperature measurement site [2]	Rate of correct core temperature measurement site according to guidelines (epitympanic in non-intubated victims not in cardiac arrest; oesophageal in victims in cardiac arrest, and/or intubated).
31	Temperature measurement (bs)	Ratio of victims for whom the temperature was measured (on site or during transport) to all victims requiring temperature measurement for decision making, according to the avalanche algorithm.
32	Core temperature measurement for CA victims (bs)	Ratio of victims in CA in whom oesophageal temperature was measured (on scene, within 15 minutes after extrication) to all victims in cardiac arrest requiring oesophageal temperature measurement for decision making, according to the avalanche algorithm.
32A	Core temperature measurement for CA victims (bs)	Ratio of victims in CA in whom oesophageal temperature was measured on scene, compared to all victims in cardiac arrest requiring oesophageal temperature measurement for decision making, according to the avalanche algorithm.
33	ECG and core T measurement (bs)	Rate of victims for whom both cardiac rhythm was analysed and temperature was measured.
109	Epitympanic temperature on site (exp)	Rate of epitympanic temperature measurement on site.
34	Appropriate thermometer (bs)	Rate of avalanche victims for whom an appropriate thermometer, epitympanic (thermistor) or oesophageal, able to measure low temperatures, was available on site.
35	Core temperature [1]	Rate of oesophageal temperature measurement among victims in cardiac arrest or intubated.
36	Severity of hypothermia [3]	Rate of hypothermic victims for whom the severity (degree) of hypothermia was estimated using the Swiss staging system or other established system, or measured using an appropriate thermometer.
37	Trauma severity reported [3]	Rate of injured victims for whom injuries were documented or injury severity score was reported.

38	Material availability (bs)	Rate of missions where all the required material to assess and treat the victim according to guidelines was available on site (e.g. airway management, adequate thermometer, ECG).
	**Patient management**	
39	Location and extrication efficiency (o.p.)	Time between location of the victim by beacon or probing and exposure of the face, divided by the burial depth.
40	Extrication time (rgp)	Time between arrival of the first rescue team to the avalanche site and exposure of the avalanche victim's face.
41	On scene time [2]	Time between arrival of the rescue team on site and departure from the site of the last victim.
42	On scene trauma care time [1] (bs)	Time between extrication of the face and departure from the site for each trauma victim.
43	Oxygenation [1] [2]	Rate of hypothermic victims receiving supplemental oxygen.
44	Advanced airway management [1] (bs)	Rate of unresponsive victims for whom advanced airway management was performed successfully (e.g. intubation, supraglottic airway device, surgical airway).
45	Airway management [2]	Rate of transported victims with a GCS<9 who underwent advanced airway management successfully (e.g. tracheal intubation, supraglottic airway device, surgical airway).
46	Intubation of cardiac arrest victims (bs)	Rate of victims in cardiac arrest who were intubated on site.
47	Normocapnia [3]	Rate of ventilated victims with a reported $ETCO_2$ value in the normocapnic range ($ETCO_2$ = 30-45 mmHg or 4-6 kPa).
101	Capnometry (exp)	Rate of victims in cardiac arrest for whom $ETCO_2$ was detected as a confirmation of ETT placement.
102	Capnometry (exp)	Rate of victims in cardiac arrest for whom initial $ETCO_2$ was <35 mmHg.
103	Capnometry (exp)	Rate of victims in cardiac arrest for whom initial $ETCO_2$ was >45 mmHg.
104	Capnography (exp)	Rate of victims in cardiac arrest for whom $ETCO_2$ was measured.
105	Hypocapnia (exp)	Rate of ventilated victims with a reported $ETCO_2$ value in the hypocapnic range ($ETCO_2$ <35 mmHg or <4 kPa).
113	Cardiac arrest after extrication (exp)	Rate of victims with cardiac arrest occurring after extrication on scene.
114	Cardiac arrest during transport (exp)	Rate of victims with cardiac arrest during transport.
48	Resuscitation start [1]	Rate of victims with temperature <30°C and patent or unknown airway for whom resuscitation (CPR) was initiated.
49	Resuscitation [1]	Rate of avalanche victims in cardiac arrest who received chest compressions and ventilation (except for victims with asystole or long burial and obstructed airway).
50	Use of mechanical CPR (bs)	Rate of use of mechanical chest compression device for victims in cardiac arrest.
51	Delayed or intermittent CPR [2] (rgp)	Rate of victims who underwent delayed or intermittent CPR according to guidelines (Gordon et. al., Resuscitation 2015: T<28°C: 5min CPR and ≤5mind without CPR, T<20°C: 5min CPR and ≤10min without CPR) compared to all victims who underwent delayed or intermittent CPR.
52	Defibrillator on site [3]	Rate of victims requiring a defibrillator or AED for whom a defibrillator or AED was available on site.
53	Defibrillation [1] [2]	Rate of victims in ventricular fibrillation and temperature<30°Cwho received a maximum of 3 defibrillation attempts.
110	Defibrillation success <30°C (exp)	Rate of victims with core temperature < 30°C with successful prehospital defibrillation
111	Defibrillation success >30°C (exp)	Rate of victims with core temperature >30°C with successful prehospital defibrillation
54	IV/IO access [2]	Rate of avalanche victims for whom IV or IO access was obtained (if feasible)
55	Epinephrine withhold [2]	Rate of victims in cardiac arrest with core temperature<30°Cfor whom epinephrine was withheld.
56	Epinephrine interval [2]	Rate of victims in cardiac arrest with core temperature between 30-35°Cfor whom epinephrine was given with longer intervals (6-10 min).
57	Post resuscitation care [2]	Rate of victims with ROSC for whom post resuscitation care was initiated using established guidelines (e.g. ERC, AHA and ESICM 2015 post-resuscitation guidelines)

58	Termination of CPR [2]	Rate of victims with temperature >30°Cand asystole and absence of reversible causes of cardiac arrest, for whom CPR was terminated after 20 min according to guidelines.
59	Termination of CPR [2]	Rate of victims with burial time >60 min with asystole and obstructed airway, for whom CPR was terminated or withheld.
60	Immobilisation CA [1]	Rate of unresponsive victims for whom spinal immobilisation was performed.
61	Cervical spine protection in unconscious victims [3]	Rate of unresponsive victims who received total body immobilisation (cervical collar and vacuum mattress or KED or spine board).
62	Trauma care [2]	Rate of patients treated according to internationally recognised prehospital trauma care guidelines (e.g. Advanced Trauma Life Support, European Trauma Course, Prehospital Trauma Life Support).
63	Insulation [1] [2] (bs)	Rate of hypothermic victims insulated with dry, low conductivity, whole body assemblies covered by a windproof and water-resistant outer shell.
64	Prehospital rewarming [1] [2]	Rate of hypothermic victims to whom an external heat source was applied to the trunk.
65	Treatment according to ERC or WMS guidelines [2]	Rate of hypothermic victims treated adequately according to European Resuscitation Council (ERC) or Wilderness Medical Society (WMS) guidelines.
66	Treatment triage in case of multiple victims [2]	Rate of victims in a multiple casualty avalanche accident who received treatment according to European Resuscitation Council (ERC) 2015 guidelines.
67	Checklist use cardiac arrest [3]	Rate of victims in cardiac arrest for whom an ICAR avalanche victim resuscitation checklist was used.
68	Checklist use complete burial (rgp)	Rate of completely buried victims for whom an ICAR avalanche victim resuscitation checklist was used.
69	Checklist use (bs)	Ratio of victims, for whom an ICAR avalanche victim resuscitation checklist was used, to all persons caught by an avalanche.
70	Completeness of checklist (bs)	Rate of ICAR avalanche victim resuscitation checklists, in which all required information was documented in accordance to the current avalanche algorithm.
71	Use of avalanche checklist (rgp) [3]	Rate of victims in cardiac arrest for whom an ICAR avalanche victim resuscitation checklist was used during the prehospital phase and transmitted to the hospital team at handover.
72	Use of avalanche checklist (rgp) [3]	Rate of victims in cardiac arrest for whom an ICAR avalanche victim resuscitation checklist was used correctly during the prehospital phase.
73	Completeness of documentation (rgp)	Rate at which all required information was documented (burial time, vital signs, airway if required, ECG if required, core temperature if required, serum potassium if required).
118	Documentation of indication of CPR stop (exp)	Ratio of victims for whom documentation included appropriate indications for CPR to be withheld or terminated, to all victims for whom CPR was withheld or terminated.
74	Dead on site [1] [2] [3]	Rate of victims declared dead on site.
	**Transport**	
75	Transport destination [1] [2]	Rate of victims who were transported to the appropriate destination according to the algorithm.
76	Mechanical chest compression device [1] [2]	Ratio of victims transported with ongoing mechanical chest compression compared to all victims who were transported with ongoing CPR.
77	Monitoring to hospital [2] (bs)	Rate of victims with continuous monitoring during transport until hospital arrival. (e.g. ECG, SpO_2, NIBP, $EtCO_2$, etc… to be defined by consensus).
78	Transport position [1]	Rate of hypothermic victims transported in a horizontal position.
79	Victim transport time (bs)	Time from departure from avalanche site to hospital arrival.
80	Adequate transport to ECLS [1]	Rate of hypothermic victims with a patent or unknown airway, with cardiac instability or a core temperature <28°C or in cardiac arrest, transported to an ECLS centre.

81	Notification of the centre [1] (bs)	Ratio of victims for whom the ECLS centre was notified prior to departure from the avalanche site, to all victims transported to an ECLS centre.
82	Procedures during transport (bs)	Rate of avalanche victims with (any) medical procedures/interventions performed during transport.
	**In-hospital management**	
83	Hospital hypothermia protocol (bs)	Hospital with internal hypothermia protocol
83A	ECLS hypothermia protocol (bs)	ECLS centre with internal hypothermia protocol
84	Appropriate rewarming [1] [2]	Appropriate in-hospital rewarming according to guidelines.
85	ECLS centre availability (bs)	Rate of avalanche victims requiring a transport to an ECLS centre who could be transported to the nearest (or first choice) ECLS centre.
86	Serum potassium [2]	Rate of victims in cardiac arrest transported to a hospital for whom serum potassium was reported.
115	Acceptance of Avalanche checklist at admission to ER (exp)	Rate of victims with full consideration and acceptance of the avalanche checklist by the emergency department consultant.
87	HOPE score [4] (rgp)	Rate of hypothermic victims in cardiac arrest for whom the HOPE (Hypothermia outcome prediction after ECLS) score was used for hospital decision making.
88	Hospital rewarming [1] [2]	Rate of hypothermic victims in cardiac arrest with a patent or unknown airway who were rewarmed to a core temperature >32°C before a decision about declaration of death was made.
89	Experienced hospital team [2]	Rate of hospital teams experienced in avalanche victim management (>5 cases per year in the emergency department).
90	Experienced hospital team [2]	Rate of hospital teams experienced in accidental hypothermia victim management (>5 cases per year in the emergency department).
91	Level of physician at hospital (bs)	Rate of victims for whom a consultant (trained and certified specialist, e.g. in emergency medicine) was in charge at hospital arrival.
92	Information transmission (bs)	Rate of victims for whom complete required information was documented on the mission report and transmitted to the hospital team on arrival.
93	In-hospital non-invasive rewarming [2]	Rate of appropriate non-invasive rewarming, according to guidelines.
94	Good outcome at hospital discharge (fully buried) (bs)	Ratio of completely buried victims with CPC score 1-2 at hospital discharge to all completely buried victims.
95	Outcome at hospital discharge (caught) (bs)	Ratio of avalanche victims with CPC 1-2 at hospital discharge to all avalanche victims.
96	Outcome at hospital discharge (CA) (bs)	Rate of victims in cardiac arrest with CPC 1-2 at hospital discharge to all victims in cardiac arrest.
116	PTSD completely buried (exp)	Rate of post-traumatic stress disorder (PTSD) among completely buried victims.
117	PTSD partially buried (exp)	Rate of post-traumatic stress disorder (PTSD) among partially buried victims.
112	Direct or indirect admission to ECLS centre (exp)	Rate of victims to a local hospital (e.g. to check serum potassium level) with immediate subsequent transport to ECLS centre.
97	Secondary transfer of victims (bs)	Rate of victims who were secondarily transferred to another hospital (within 24 hours after the first admission).
98	Impact of hypothermia staging on destination (exp)	Rate of victims for whom the temperature or hypothermia staging (according to the Revised Swiss System) was used to determine the destination hospital (e.g. ECLS centre or non-ECLS centre).
99	Trained persons at the medical dispatch centre (exp)	Rate of trained persons handling emergency calls for avalanche accidents.
100	Victims receiving ECMO or CPB (exp)	Rate of victims in CA who received ECMO or CPB (ECLS) according to guidelines.

1. Brugger H, Durrer B, Elsensohn F, Paal P, Strapazzon G, Winterberger E, Zafren K, Boyd J: **Resuscitation of avalanche victims: Evidence-based guidelines of the international commission for mountain emergency medicine (ICAR MEDCOM): intended for physicians and other advanced life support personnel**. *Resuscitation* 2013, **84**(5):539-546.
2. Truhlar A, Deakin CD, Soar J, Khalifa GE, Alfonzo A, Bierens JJ, Brattebo G, Brugger H, Dunning J, Hunyadi-Anticevic S *et al*: **European Resuscitation Council Guidelines for Resuscitation 2015: Section 4. Cardiac arrest in special circumstances**. *Resuscitation* 2015, **95**:148-201.
3. Van Tilburg C, Grissom CK, Zafren K, McIntosh S, Radwin MI, Paal P, Haegeli P, Smith WW, Wheeler AR, Weber D *et al*: **Wilderness Medical Society Practice Guidelines for Prevention and Management of Avalanche and Nonavalanche Snow Burial Accidents**. *Wilderness Environ Med* 2017, **28**(1):23-42.
4. Pasquier M, Hugli O, Paal P, Darocha T, Blancher M, Husby P, Silfvast T, Carron PN, Rousson V: **Hypothermia outcome prediction after extracorporeal life support for hypothermic cardiac arrest victims: The HOPE score**. *Resuscitation* 2018, **126**:58-64.

Supplementary File S3: Quality indicator selection by the expert group during the consensus meeting

**QIs removed (n=3)**
Established post resuscitation care (e.g. ERC, AHA and ESICM 2015 post-resuscitation guidelines) initiated for victims with ROSC.
Victims treated adequately according to ERC (European Resuscitation Council) 2015 and WMS (Wilderness Medical Society) guidelines.
Patients are transported to the adequate destination according to the algorithm.
**QI added (n=1)**
Ratio of victims whose core temperature was measured at the avalanche site to avalanche victims for whom core temperature should have been measured.

Supplementary File S4: Data required to calculate the quality indicators (QIs) for avalanche victim management and rescue.

Quality indicator	Data required to calculate the QI
**Prior to the avalanche rescue mission**	
Time between alarm to dispatch centre and on-scene arrival (bs)	Time of alarm to the dispatch center<br>Arrival time of first organized rescue team on scene (helicopter, terrestrial, or ski patrol)
Time between accident and on-scene arrival (bs)	Time of accident<br>Arrival time of first organized rescue team on scene (helicopter, terrestrial, or ski patrol)
Burial time (1, 2) (bs)	Time of accident<br>Time of exposure of the face
CPR by bystanders (2)	Degree of burial (complete or partial)<br>Signs of life at extrication (yes/no)<br>CPR by comrades/bystanders or first responders (ski patrol) CPR directly after extrication (yes/no)
**Patient assessment**	
Airway patency (1, 2)	Burial time<br>Degree of burial (complete or partial)<br>Assessment of airway patency at face exposure (patent, not patent, or unknown)
Documentation of air pocket (3)	Degree of burial (complete or partial)

	Documentation of the existence of an air pocket (yes/no)
ECG monitoring (1)	Presence of signs of life (yes/no)<br>Clear signs of death (yes/no) (clear signs of death include airway obstructed with packed snow AND burial time >60min AND Asystole, decapitation, whole body frozen.)<br>ECG monitoring performed on site (yes/no)
Site of temperature measurement (2)	Temperature measurement on site (yes/no)<br>Temperature measurement site (epitympanic, esophageal, other)<br>Victim in cardiac arrest (yes/no)
Temperature measurement on site (2)	Temperature measurement on site (yes/no)<br>Temperature measurement required according to algorithm? (yes/no)
**Patient management**	
Airway management (2)	Advanced airway management attempted (yes/no)<br>Success of advanced airway management (yes/no)<br>Transport to hospital (yes/no)
Circumrescue collapse (exp)	Witnessed cardiac arrest during rescue (yes/no)<br>Victim in cardiac arrest at first contact (yes/no)
CPR initiation after long burial (1)	Burial duration<br>Core temperature<br>Signs of life<br>Clear signs of death<br>CPR initiated (yes/no)
Overall CPR (1)	Victim in cardiac arrest (yes/no)<br>CPR initiated (yes/no)<br>Ventilation (yes/no)<br>Burial duration<br>Assessment of airway patency (patent, not patent, unknown)
Termination of CPR after short burial (2)	Initial cardiac rhythm asystole (yes/no)<br>Core temperature<br>Burial duration<br>Victim in cardiac arrest at extrication (yes/no)<br>Reversible causes of cardiac arrest (yes/no)<br>Number of victims in cardiac arrest at the avalanche accident<br>CPR terminated on site (yes/no)<br>Termination of resuscitation /CPR according to guidelines (yes/no)
Termination of CPR after long burial (2)	Victim in cardiac arrest (yes/no)<br>Burial duration<br>Initial cardiac rhythm asystole (yes/no)<br>CPR initiated (yes/no)<br>Resuscitation or CPR terminated on site (yes/no)<br>Assessment of airway patency (patent, not patent, unknown)

Insulation (1, 2) (bs)	Temperature measurement on site (yes/no)<br>Core temperature<br>Clinical stage of hypothermia according to the Revised Swiss System (4)<br>Whole body insulation (yes/no)
Use of avalanche checklist (3)<br>(rgp)	Presence of signs of life (yes/no)<br>Victim in cardiac arrest (yes/no)<br>Avalanche victim resuscitation checklist used (yes/no)<br>Avalanche victim resuscitation checklist transmitted to the hospital (yes/no)
Completeness of documentation (rgp)	All required information documented (burial duration, signs of life, airway patency if required, ECG if required, core temperature if required, serum potassium if required) (yes/no)
**Transport**	
Appropriate transport to ECLS (1)	Core temperature<br>Clinical stage of hypothermia according to the Revised Swiss System (4)<br>Type of hospital (ECLS vs non-ECLS) to which the patient was transported
**In-hospital management**	
Appropriate rewarming (1, 2)	Type of in-hospital rewarming (ECLS, non-invasive, other)
Serum potassium (2)	Serum potassium measurement (yes/no)
Hospital rewarming (1, 2)	Death declared in hospital before rewarming to core-temperature >32° (yes/no)
Patients to ECMO or CPB (exp)	Victim in cardiac arrest at hospital arrival (yes/no)<br>In-hospital ECLS rewarming (yes/no)

1. Brugger H, Durrer B, Elsensohn F, Paal P, Strapazzon G, Winterberger E, et al. Resuscitation of avalanche victims: Evidence-based guidelines of the international commission for mountain emergency medicine (ICAR MEDCOM): intended for physicians and other advanced life support personnel. Resuscitation. 2013;84(5):539-46.
2. Truhlar A, Deakin CD, Soar J, Khalifa GE, Alfonzo A, Bierens JJ, et al. European Resuscitation Council Guidelines for Resuscitation 2015: Section 4. Cardiac arrest in special circumstances. Resuscitation. 2015;95:148-201.
3. Van Tilburg C, Grissom CK, Zafren K, McIntosh S, Radwin MI, Paal P, et al. Wilderness Medical Society Practice Guidelines for Prevention and Management of Avalanche and Nonavalanche Snow Burial Accidents. Wilderness Environ Med. 2017;28(1):23-42.
4. Musi ME, Sheets A, Zafren K, Brugger H, Paal P, Holzl N, et al. Clinical staging of accidental hypothermia: The Revised Swiss System: Recommendation of the International Commission for Mountain Emergency Medicine (ICAR MedCom). Resuscitation. 2021;162:182-7.
